# Comparison of molecular profile in triple-negative inflammatory and non-inflammatory breast cancer not of mesenchymal stem-like subtype

**DOI:** 10.1371/journal.pone.0222336

**Published:** 2019-09-18

**Authors:** Yohei Funakoshi, Ying Wang, Takashi Semba, Hiroko Masuda, David Hout, Naoto T. Ueno, Xiaoping Wang

**Affiliations:** 1 Morgan Welch Inflammatory Breast Cancer Research Program and Clinic, The University of Texas MD Anderson Cancer Center, Houston, Texas, United States of America; 2 Section of Translational Breast Cancer Research, The University of Texas MD Anderson Cancer Center, Houston, Texas, United States of America; 3 Department of Breast Medical Oncology, The University of Texas MD Anderson Cancer Center, Houston, Texas, United States of America; 4 Department of Biostatistics, The University of Texas MD Anderson Cancer Center, Houston, Texas, United States of America; 5 Insight Genetics, Inc., Nashville, Tennessee, United States of America; University of South Alabama Mitchell Cancer Institute, UNITED STATES

## Abstract

**Background:**

Inflammatory breast cancer (IBC) is an aggressive form of breast cancer. The triple-negative subtype of IBC (TN-IBC) is particularly aggressive. Identification of molecular differences between TN-IBC and TN-non-IBC may help clarify the unique clinical behaviors of TN-IBC. However, our previous study comparing gene expression between TN-IBC and TN-non-IBC did not identify any TN-IBC-specific molecular signature. Lehmann et al recently reported that the mesenchymal stem-like (MSL) TNBC subtype consisted of infiltrating tumor-associated stromal cells but not cancer cells. Therefore, we compared the gene expression profiles between TN-IBC and TN-non-IBC patient samples not of the MSL subtype.

**Methods:**

We classified 88 TNBC samples from the World IBC Consortium into subtypes according to the Vanderbilt classification and Insight TNBCtype, removed samples of MSL and unstable subtype, and compared gene expression profiles between the remaining TN-IBC and TN-non-IBC samples.

**Results:**

In the Vanderbilt analysis, we identified 75 genes significantly differentially expressed between TN-IBC and TN-non-IBC at an FDR of 0.2. In the Insight TNBCtype analysis, we identified 81 genes significantly differentially expressed between TN-IBC and TN-non-IBC at an FDR of 0.4. In both analyses, the top canonical pathway was “Fc Receptor-mediated Phagocytosis in Macrophages and Monocytes”, and the top 10 differentially regulated genes included *PADI3* and *MCTP1*, which were up-regulated, and *CDC42EP3*, *SSR1*, *RSBN1*, and *ZC3H13*, which were downregulated.

**Conclusions:**

Our data suggest that the activity of macrophages might be enhanced in TN-IBC compared with TN-non-IBC. Further clinical and preclinical studies are needed to determine the cross-talk between macrophages and IBC cells.

## Introduction

Inflammatory breast cancer (IBC) is a highly aggressive form of breast cancer and is associated with higher rates of recurrence and metastasis and a lower survival rate than non-IBC [1]. Several molecular changes have been found to contribute to the aggressiveness of IBC, including loss of *WISP3* and overexpression of RhoC GTPase [2], E-cadherin [3], translation initiation factor eIF4GI [4], and tazarotene-induced gene 1 [5]. Our research group also reported that EGFR signaling promoted inflammation and cancer stem-like cell activity in IBC [6], and the EGFR pathway is a promising therapeutic target for patients with triple-negative IBC (TN-IBC) [7, 8]. Recent studies suggested that immune cells in the tumor microenvironment, especially macrophages, play a major role in regulating the malignant phenotype of IBC [9, 10]. Although these findings have improved our understanding of the molecular mechanisms underlying the aggressive behavior of IBC, unique genes that contribute to the aggressiveness of IBC have not yet been identified. Identification of such genes is critical to facilitate development of US Food and Drug Administration-approved targeted therapies for this disease.

Triple-negative breast cancer (TNBC) is characterized by lack of expression of estrogen receptor, progesterone receptor, and human epidermal growth factor receptor 2. Patients with TNBC have a worse prognosis than other breast cancer patients. Generally, 10% to 20% of patients with non-IBC have TNBC (TN-non-IBC), whereas 20% to 40% of patients with IBC have TN-IBC [11–13]. It has been speculated that the high percentage of TNBC among patients with IBC may be associated with the more aggressive clinical course and decreased overall and breast cancer-specific survival of patients with IBC [14].

A research group at Vanderbilt University reported that TNBC can be classified into 7 molecular subtypes on the basis of differential gene expression and gene ontologies, including basal-like 1 (BL1), basal-like 2 (BL2), immunomodulatory (IM), mesenchymal (M), mesenchymal stem-like (MSL), luminal androgen receptor (LAR), and unstable (UNS) [15]. In a previous study, our research group identified these 7 TNBC subtypes in patients with TN-IBC [16]. Because IBC is more aggressive than non-IBC, we hypothesized that the distribution of the 7 TNBC subtypes differs between TN-IBC and TN-non-IBC. However, our findings did not support this hypothesis: we found no significant difference in the distribution between TN-IBC and TN-non-IBC. Furthermore, comparison of gene expression profiles between patients with TN-IBC and TN-non-IBC did not identify any promising molecular signatures specific to the TN-IBC group. This study led us to conclude that not only tumor cells but also their microenvironment and other factors, such as inflammation, immune pathways, and mutations, may contribute to the specific biology of TN-IBC.

Recently, the Vanderbilt research group reported that the MSL gene expression signature was contributed from infiltrating tumor-associated stromal cells but not breast cancer cells [17]. Because surgical specimens may contain large amounts of stromal cells and normal cells, TNBC specimens of MSL subtype would provide inappropriate information for analysis. Therefore, in a new attempt to identify cancer-specific genes that contribute to the aggressiveness of TN-IBC, we decided to compare the gene expression profiles between TN-IBC and TN-non-IBC patient samples not of the MSL subtype.

## Material and methods

### Patients

This study was approved by the Institutional Review Board of The University of Texas MD Anderson Cancer Center (protocol number PA15-0954). We retrospectively analyzed gene expression profiles and clinical data of all 88 patients with TNBC with known IBC status (39 patients with IBC and 49 with non-IBC) from the World IBC Consortium dataset, contributed by MD Anderson Cancer Center, Houston, TX; General Hospital Sint-Augustinus, Antwerp, Wilrijk, Belgium; and Institut Paoli-Calmettes, Marseille, France [18]. Patients at each site gave informed consent for voluntary participation, and the study was approved by the institutional review boards of the 3 participating centers. IBC was identified according to the consensus diagnostic criteria [12]. TNBC was diagnosed according to gene expression profiling as reported in our previous paper [16].

TNBC was divided into 7 molecular subtypes (BL1, BL2, M, MSL, IM, LAR, and UNS) according to the Vanderbilt classification [15]. TNBC was also divided into 6 molecular subtypes (BL1, BL2, M, MSL, LAR, and UNS) according to Insight TNBCtype (Insight Genetics, Inc., Nashville, TN, USA), a new assay for TNBC subtyping that reduces the number of genes from the original 2188 genes described by Lehmann et al to 101 genes, including control housekeeping genes [19, 20]. IM subtype was removed in Insight TNBCtype because IM subtype likely reflects infiltrating lymphocytes within tumor. After subtyping was complete, the samples classified as MSL and UNS were excluded. Samples of MSL and UNS subtype were not included in the statistical analyses.

### Statistical analysis

The data processing and statistical analyses were performed in R version 3.3.0 [21]. Differences between IBC and non-IBC at the gene expression level were examined using feature-by-feature 2-sample t tests followed by a beta-uniform mixture model to adjust for multiple comparisons [22]. Genes with significantly different expression between TN-IBC and TN-non-IBC were counted at different false discovery rates (FDRs).

To identify pathways that may contribute to the aggressiveness of TN-IBC, we performed Ingenuity Pathway Analysis of genes differentially expressed between non-MSL TN-IBC and non-MSL TN-non-IBC in both the Vanderbilt and Insight TNBCtype analyses.

To study the association of genes of interest with overall survival, Kaplan-Meier plots were generated for patients with TNBC in The Cancer Genome Atlas (TCGA) (N = 115) with high and low gene expression (http://cancergenome.nih.gov/). For each specific gene, a log-rank test was performed to compare the survival functions between patients with gene expression above and below the median level (“high” and “low” gene expression groups, respectively).

### Western blotting

For Western blotting, cells were lysed in lysis buffer [50 mM Tris (pH 7.5), 150 mM NaCl, 1% NP40, 0.5% sodium deoxycholate acid, 0.1% sodium dodecyl sulfate, protease inhibitor cocktail (Bimake.com, Houston, TX), and phosphatase inhibitor cocktail (Bimake.com)]. Proteins were fractioned by SDS-PAGE on 4% to 12% NuPAGE Bis-Tris gels (Thermo Fisher Scientific, Waltham, MA) and transferred to Immun-Blot PVDF membrane (Bio-Rad, Hercules, CA). Proteins of interest were probed using anti-PADI3 antibody (rabbit polyclonal, Sigma-Aldrich, St. Louis, MO) and anti-β-actin antibody (Sigma-Aldrich).

### siRNA transfection

Using Lipofectamine RNAiMAX (Thermo Fisher Scientific) according to the manufacturer’s instructions, 4 × 10^5^ SUM149 cells were transfected with ON-TARGETplus non-targeting siRNA (Dharmacon, Lafayette, CO) or SMARTpool: ON-TARGETplus PADI3 siRNA (a mixture of 4 designed siRNAs targeting *PADI3*; Dharmacon) at a final siRNA concentration of 16.7 μM.

### Cell proliferation assay

Seventy-two hours after siRNA transfection, 1 × 10^5^ cells were seeded in complete medium in a 12-well plate and incubated for 72 h. Viable cells were counted using the trypan blue dye exclusion method by Vi-CELL XR (Beckman Coulter, Brea, CA).

### Anchorage-independent growth

Seventy-two hours after siRNA transfection, 8 × 10^3^ cells were resuspended in 0.38% agarose medium and then plated in 12-well plates coated with solidified 0.75% agarose medium. Three weeks later, colonies greater than 50 μm in diameter were counted using the Gel Count system (Oxford Optronix Ltd., Milton Park, Abingdon, UK).

## Results

### Genes differentially expressed between non-MSL TN-IBC and non-MSL TN-non-IBC

The characteristics of the 88 patients with TNBC were described in our previous publication [23].

**Fig 1:**
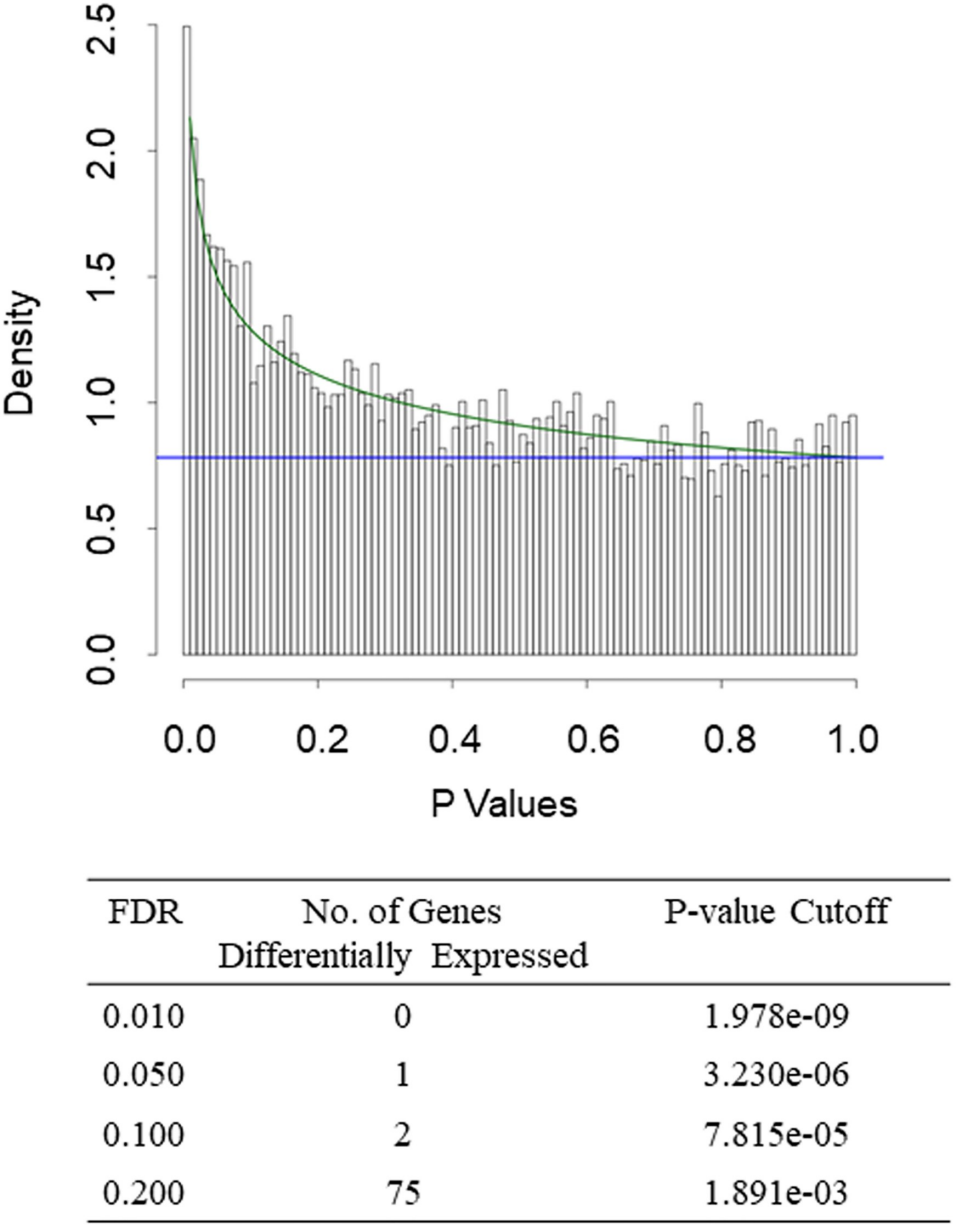
Gene expression in IBC versus non-IBC in 70 patients with non-MSL TNBC according to the Vanderbilt classification. Top, Histogram of *P* values from 2-sample *t* tests for gene expression in TN-IBC vs. TN-non-IBC. The overlaid curve is the fitted BUM model. Bottom, Counts of differentially expressed genes with various FDR cutoffs.

Classification of samples from the 88 patients with TNBC (39 with IBC and 49 with non-IBC) according to the Vanderbilt classification [15] revealed 11 samples (8 TN-IBC and 3 TN-non-IBC) with MSL subtype and 7 samples (2 TN-IBC and 5 TN-non-IBC) with UNS subtype. After excluding these samples, we had 29 TN-IBC samples and 41 TN-non-IBC samples. Comparison of the gene expression profiles between these non-MSL TN-IBC and TN-non-IBC samples revealed 75 genes differentially expressed at an FDR of 0.2 (Fig 1 and S1 Table).

**Fig 2:**
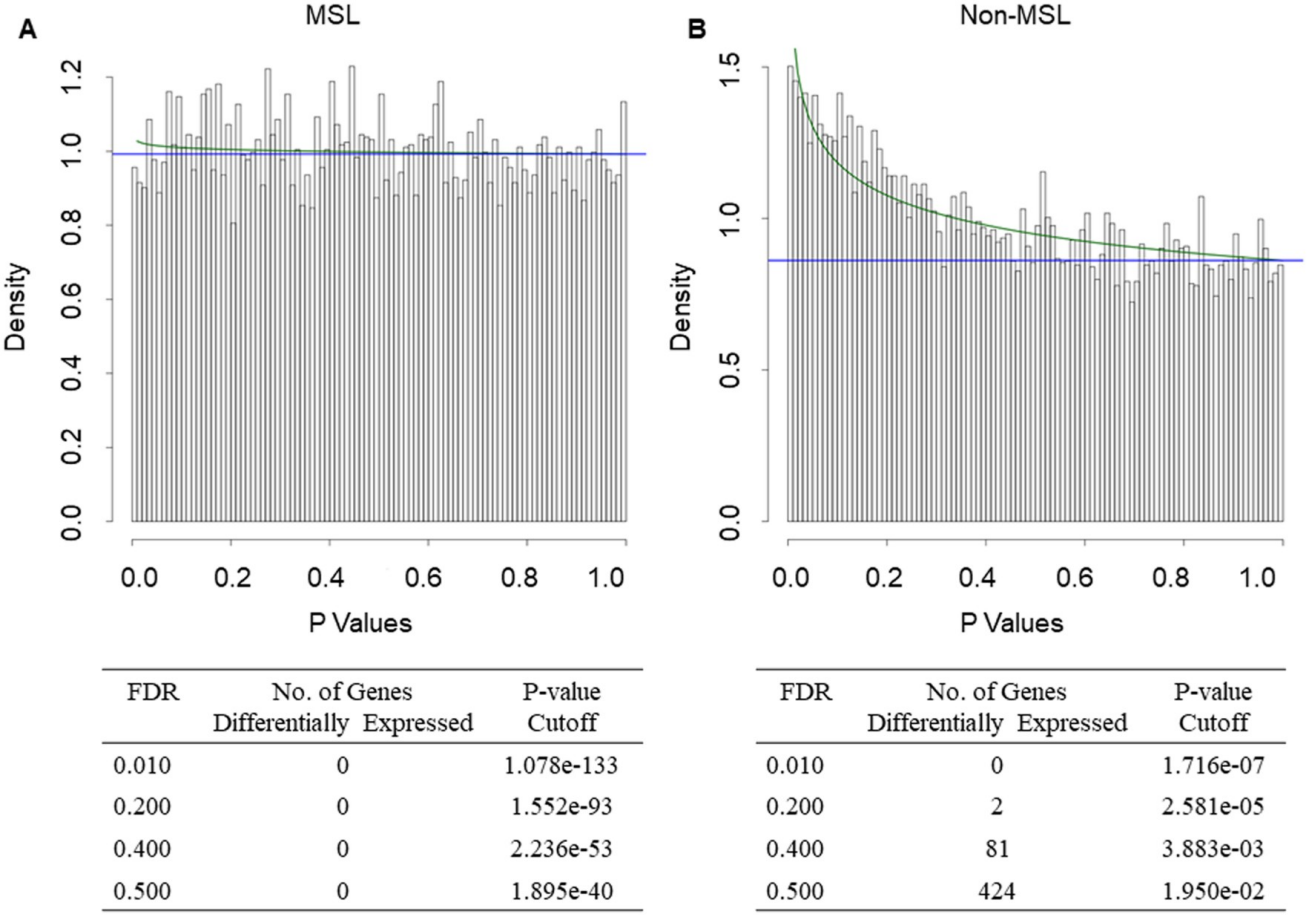
Gene expression in IBC versus non-IBC in (A) 52 patients with non-MSL TNBC and (B) 31 patients with MSL TNBC according to Insight TNBCtype. Top of each panel, Histogram of P values from 2-sample *t* test for gene expression in TN-IBC versus TN-non-IBC. The overlaid curves are the fitted BUM models. Bottom of each panel, Counts of differentially expressed genes with various FDR cutoffs.

Classification of samples from the 88 patients with TNBC according to Insight TNBCtype revealed 31 samples (16 TN-IBC and 15 TN-non-IBC) with MSL subtype and 5 samples (1 TN-IBC and 4 TN-non-IBC) with UNS subtype. After excluding these samples, we had 22 TN-IBC samples and 30 TN-non-IBC samples. Comparison of the gene expression profiles between these non-MSL TN-IBC and TN-non-IBC samples revealed 81 genes differentially expressed at an FDR of 0.4 (Fig 2A and S2 Table). We also compared the gene expression profiles between the MSL TN-IBC and MSL TN-non-IBC samples and did not identify any significantly differentially expressed genes (Fig 2B).

### Genes and signaling pathways that may contribute to the aggressiveness of TN-IBC

Ingenuity Pathway Analysis showed that the top canonical pathway in both analyses was “Fc Receptor-mediated Phagocytosis in Macrophages and Monocytes” (Table 1).

**Table 1:** Top 5 canonical pathways derived from Ingenuity Pathway Analysis gene ontology algorithms^a^.

Canonical pathway	*P* value	Overlap%^b^
*Vanderbilt*		
Fc Receptor-mediated Phagocytosis in Macrophages and Monocytes	1.72E-04	4.3 (4/93)
Systemic Lupus Erythematosus Signaling	4.58E-03	1.8 (4/225)
Paxillin Signaling	4.69E-03	2.7 (3/113)
Molecular Mechanisms of Cancer	5.15E-03	1.3 (5/374)
Ethanol Degradation II	5.46E-03	5.4 (2/37)
*TNBCtype*		
Fc Receptor-mediated Phagocytosis in Macrophages and Monocytes	3.81E-03	3.2 (3/93)
Heme Biosynthesis from Uroporphyrinogen-III I	1.34E-02	25.0 (1/4)
Protein Citrullination	1.67E-02	20.0 (1/5)
RAR Activation	2.62E-02	1.6 (3/190)
VDR/RXR Activation	2.84E-02	2.6 (2/78)

^a^ Results are based on 75 differentially expressed genes identified in the analysis based on the Vanderbilt classification and 81 differentially expressed genes identified in the analysis based on Insight TNBCtype.

^b^ The overlap in Canonical pathways represents the ratio of analysis ready dataset molecules over the total molecules present in the particular Canonical Pathway.

We further identified the top 10 genes differentially expressed between non-MSL TN-IBC and non-MSL TN-non-IBC (Table 2). In both the Vanderbilt and Insight TNBCtype analyses, the top 10 differentially regulated genes included *PADI3* and *MCTP1*, which were up-regulated, and *CDC42EP3*, *SSR1*, *RSBN1*, and *ZC3H13*, which were down-regulated. The functions of these 6 genes are summarized in Table 3. Analysis of the association between the expression of these genes and overall survival in 115 patients with TNBC from the TCGA database did not identify any significant associations (S1 Fig).

**Table 2:** Top 10 up-regulated and down-regulated genes in non-MSL TN-IBC versus non-MSL TN-non-IBC^a^.

Up-regulated in TN-IBC		Down-regulated in TN-IBC	
Molecule	Log ratio	Molecule	Log ratio
*Vanderbilt*			
*PADI3	0.604	*SERPINE2*	-0.941
*ARF6*	0.574	*CNN3*	-0.836
*MCTP1	0.551	*CDC42EP3	-0.622
*TFPI*	0.471	*NME7*	-0.535
*ZFP36*	0.399	*SSR1	-0.528
*CLEC1A*	0.351	*SSR3*	-0.527
*PAK4*	0.344	*RSBN1	-0.466
*NOTCH4*	0.309	*SNRNP40*	-0.461
*EFCC1*	0.298	*CRK*	-0.443
*PLXND1*	0.282	*ZC3H13	-0.442
*TNBCtype*			
*PADI3	0.750	*CDC42EP3	-0.752
*MCTP1	0.675	*LOC730101*	-0.682
*TCF4*	0.671	*SSR1	-0.652
*RPL37A*	0.670	*TARDBP*	-0.609
*COA1*	0.647	*ZC3H13	-0.609
*MICAL2*	0.558	*PRKD3*	-0.584
*FPR1*	0.538	*KDM3B*	-0.559
*LPIN2*	0.496	*COPG1*	-0.551
*MERTK*	0.439	*RSBN1	-0.548
*MAPKAPK3*	0.364	*DOPEY1*	-0.510

^a^ Results are based on 75 differentially expressed genes identified in the analysis based on the Vanderbilt classification and 81 differentially expressed genes identified in the analysis based on Insight TNBCtype.

* Genes that were differentially regulated in both the Vanderbilt and Insight TNBCtype analyses.

**Table 3:** Main functions of genes differentially expressed between non-MSL TN-IBC and non-MSL TN-non-IBC in both the Vanderbilt and Insight TNBCtype analyses.

Gene symbol	Gene name	Main functions
*PADI3*	Peptidyl arginine deiminase 3	Protein citrullination in hair follicles, keratinocytes, and macrophages
*MCTP1*	Multiple C2 and transmembrane domain containing 1	Unknown
*CDC42EP3*	CDC42 effector protein 3	Organization of actin cytoskeleton; regulation of tumor-associated fibroblasts
*SSR1*	Signal sequence receptor subunit 1	Part of a glycosylated endoplasmic reticulum membrane receptor
*RSBN1*	Round spermatid basic protein 1	Unknown
*ZC3H13*	Zinc finger CCCH-type containing 13	Regulation of mRNA degradation

**Fig 3:**
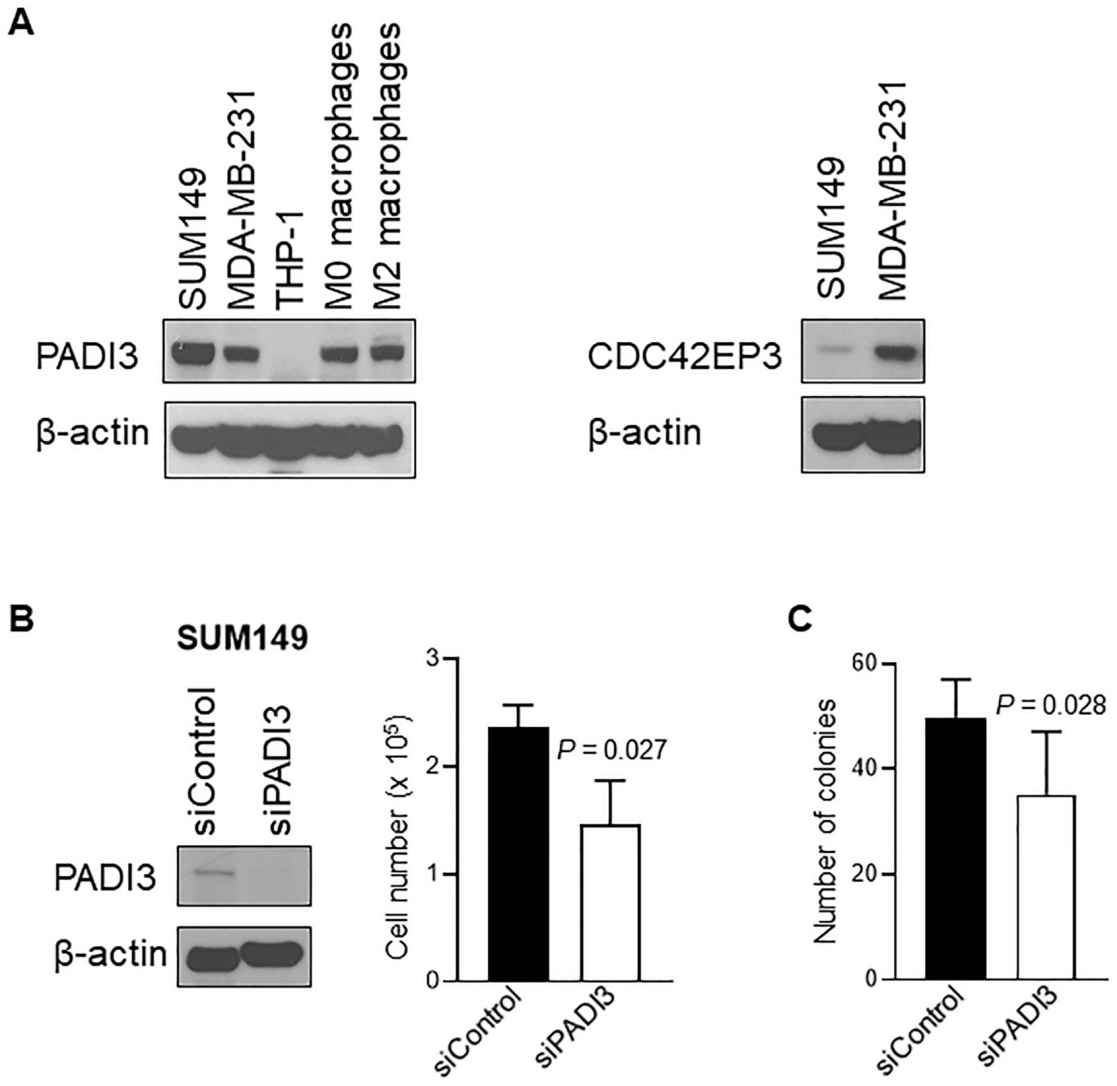
Analysis of PADI3 and CDC42EP3. **(A) The expression of PADI3 and CDC42EP3 in breast cancer cell lines and macrophages**. Expressions of PADI3 in SUM149 (TN-IBC), MDA-MB-231 (TN-non-IBC) cells, THP-1 (monocytes), M0 macrophages (THP-1-derived immature macrophages) and M2 macrophages (THP-1–derived M2 macrophages) were analyzed with Western blotting. Expressions of CDC42EP3 in SUM149 and MDA-MB231 were analyzed with Western blotting. **(B) PADI3 knockdown suppresses cell growth in SUM149**. Left panel: The expression of PADI3 in SUM149 cells was depleted with siRNA and the expression of PADI3 in siControl and siPADI3 was analyzed with western blot. Right panel: Proliferation of SUM149 cells transfected with siControl and siPADI3 was measured by Trypan blue exclusion assay. Bars, SD. **(C) PADI3 knockdown suppresses anchorage-independent growth of SUM149 cells**. Bars, SD.

Next we examined the expression of PADI3 and CDC42EP3 in TN-IBC SUM149 and TN-non-IBC MDA-MB-231 cells using Western blotting. As shown in Fig 3A, PADI3 protein expression was higher in SUM149 cells than in MDA-MB-231 cells. In contrast, CDC42EP3 expression was lower in SUM149 cells than in MDA-MB-231 cells. These results validated the findings regarding differential gene expression of *PADI3* and *CDC42EP3* in TN-IBC and TN-non-IBC (Table 2). It has been reported that PADI3 is related to macrophages [24], so we examined the expression level of PADI3 in THP-1-derived M0 and M2 macrophages. Our data showed that both M0 and M2 macrophages expressed PADI3 protein (Fig 3A).

To further understand the function of PADI3 in TN-IBC, we depleted the expression of PADI3 in TN-IBC SUM149 cells using siRNA and measured cell proliferation. As shown in Fig 3B, compared to siControl, siPADI3 reduced the proliferation of SUM149 cells by 38% (*P* = 0.027). We also examined whether depletion of PADI3 affects anchorage-independent growth of SUM149 cells. The number of colonies formed by siPADI3 was less than that of siControl (Fig 3C, *P* = 0.028). These results suggested that PADI3 may play a role in the growth of TN-IBC cells.

## Discussion

Compared with our previous study, our present study identified more genes that were differentially expressed in TN-IBC versus TN-non-IBC: 75 genes in the analysis based on the Vanderbilt classification (vs. 0 in our previous study) at an FDR of 0.2 [16] and 81 genes in the analysis based on Insight TNBCtype (vs. 38 in our previous study) at an FDR of 0.4 [16]. We therefore decided to analyze the overlapping genes between the 2 sets of results. We recognize that these genes were identified at a high FDR, which limited statistical power. However, this alternative approach allowed us to identify molecules and signaling pathways that may contribute to the aggressiveness of IBC. Indeed, we were able to validate the higher expression of PADI3 in TN-IBC SUM149 cells than in TN-non-IBC MDA-MB-231 cells (Fig 3A).

Analysis of the 31 MSL samples (16 TN-IBC and 15 TN-non-IBC) classified according to Insight TNBCtype did not detect any gene differentially expressed between TN-IBC and TN-non-IBC even at an FDR of 0.5 (Fig 2B).

The functions of the 6 genes that were among the top 10 differentially regulated genes in both the Vanderbilt and Insight TNBCtype analyses are summarized in Table 3. Among these genes, *PADI3* (peptidyl arginase deiminase 3) and *CDC42EP3* (CDC42 effector protein 3) are of particular interest because of their roles in macrophages and tumor biology. PADI3 is responsible for the citrullination of proteins in a calcium-dependent manner [25]. It is known to be distributed in hair follicles and keratinocytes, and a recent report indicated that PADI3 was strongly stained in macrophages (CD68-positive cells) in rheumatoid nodules and synovial tissue [24]. Consistent with that report, we found expression of PADI3 protein in THP-1 polarized M0 and M2 macrophages (Fig 3A). Furthermore, the higher expression of PADI3 in TN-IBC SUM149 cells than in TN-non-IBC MDA-MB-231 cells (Fig 3A) and the finding depletion of PADI3 reduced the proliferation of SUM149 cells (Fig 3B) indicated that PADI3 may play an important role in promoting the aggressiveness of IBC. CDC42EP3 is a downstream molecule of CDC42 and is involved in organization of the actin cytoskeleton [26, 27]. A recent study suggested that CDC42EP3 is a key regulator of cancer-associated fibroblasts. The expression of CDC42EP3 potentiates cellular responses to mechanical stimulation, leading to signaling and transcriptional adaptations required for activation of the cancer-associated fibroblast phenotype [28]. However, in our study, *CDC42EP3* gene expression was downregulated in TN-IBC samples, which suggests that CDC42EP3-regulated cancer-associated fibroblasts may not contribute to the aggressiveness of TN-IBC. Consistent with our gene analysis data, we found that protein expression of CDC42EP3 was lower in TN-IBC SUM149 cells than in TN-non-IBC MDA-MB-231 cells (Fig 3A). The functions of the other genes that were differentially expressed between non-MSL TN-IBC and non-MSL TN-non-IBC in both the Vanderbilt and Insight TNBCtype analyses (*MCTP1*, *SSR1*, *RSBN1*, and *ZC3H13*) are still poorly understood and need to be further studied.

Our findings that Fc Receptor-mediated Phagocytosis in Macrophages and Monocytes was the top canonical pathway in both datasets and *PADI3* was the top up-regulated gene suggest that the activity of macrophages may contribute to the specific biology of TN-IBC. Recent studies have indicated that the tumor microenvironment is a critical driver of the IBC clinical phenotype. It has been reported that macrophages enhance the migration of IBC cells through RhoC GTP signaling [9], and macrophages isolated from IBC patient samples secrete chemotactic cytokines that may increase IBC tumor cell dissemination and metastasis [18]. Woodward’s group showed that macrophage-educated mesenchymal stem cells promote the invasion, mammosphere formation, and IL-6 secretion of IBC cells [10]. We also previously found that high expression of CD163, an M2 macrophage marker, correlated with short disease-free survival of patients with IBC (unpublished data). Our current study further supports the critical role of the tumor microenvironment in promoting IBC progression. Further study is needed to determine the role of the differentially expressed genes that we identified in the aggressiveness of TN-IBC.

In conclusion, our study identified molecules and signaling pathways related to the activity of macrophages that may contribute to the unique biology of TN-IBC. These candidates need to be further validated in preclinical and clinical studies.

## Supporting information

S1 Fig Association between the expression of (A) *PADI3*, (B) *MCTP1*, (C) *CDC42EP3*, (D) *SSR1*, (E) *RSBN1*, and (F) *ZC3H13* and overall survival of patients with TNBC using TCGA data. (TIF)

S1 Table Seventy-five genes differentially expressed between non-MSL TN-IBC and non-MSL TN-non-IBC at an FDR of 0.2 based on the Vanderbilt classification. (DOCX)

S2 Table Eighty-one genes differentially expressed genes between non-MSL TN-IBC and non-MSL TN-non-IBC at an FDR of 0.4 based on Insight TNBCtype. (DOCX)

S3 Table 88 patients clinical data. (XLSX)
