# Supplementary figures and images for: Comparison of molecular profile in triple-negative inflammatory and non-inflammatory breast cancer not of mesenchymal stem-like subtype
Source: PLoS One. 2019 Sep 18;14(9):e0222336. doi: 10.1371/journal.pone.0222336 (PMC6750603; doi:10.1371/journal.pone.0222336)

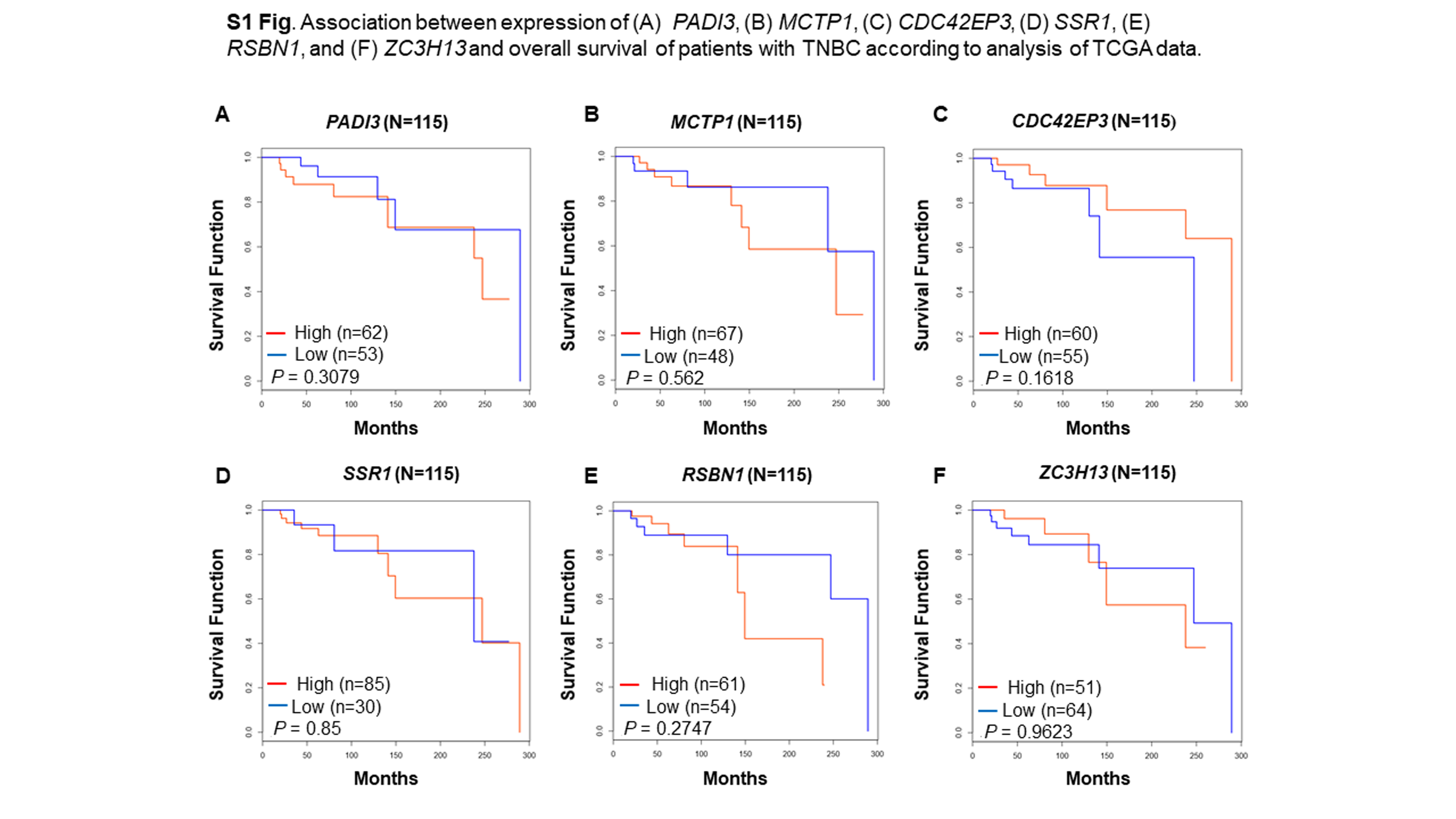

Supplement: S1 Fig — (TIF) [file pone.0222336.s001.tif]
