# Supplementary material for: Comparison of molecular profile in triple-negative inflammatory and non-inflammatory breast cancer not of mesenchymal stem-like subtype
Source: PLoS One. 2019 Sep 18;14(9):e0222336. doi: 10.1371/journal.pone.0222336 (PMC6750603; doi:10.1371/journal.pone.0222336)
Supplement: S1 Table — (DOCX) [file pone.0222336.s002.docx]

**S1 Table. Seventy-five genes differentially expressed at an FDR of 0.2 between non-MSL TN-IBC and non-MSL TN-non-IBC based on the Vanderbilt classification.**

| **Gene** | **Log2FC.IBC** | ***P* value** |
| --- | --- | --- |
| *ADH6* | 0.141287 | 0.000869 |
| *ADRBK1* | 0.240493 | 0.001347 |
| *AFFX-M278* | 0.266126 | 0.001152 |
| *ALMS1* | -0.3337 | 0.0009 |
| *APH1A* | -0.3941 | 0.000595 |
| *BGLAP* | -0.32293 | 0.000338 |
| *BTNL2* | 0.182449 | 0.000288 |
| *C11orf57* | -0.32451 | 0.00063 |
| *C2CD2L* | 0.260178 | 0.00027 |
| *CBL* | 0.164372 | 0.001714 |
| *CCDC48* | 0.298248 | 0.001355 |
| *CDC42EP3* | -0.62239 | 0.001615 |
| *CLEC1A* | 0.350707 | 0.000325 |
| *CLUAP1* | -0.37496 | 0.00038 |
| *CNN3* | -0.83607 | 0.000294 |
| *CYP3A4* | 0.131855 | 0.000928 |
| *DEFB4A* | 0.215851 | 0.001437 |
| *DHX57* | -0.32257 | 0.000621 |
| *DOPEY1* | -0.3352 | 0.000264 |
| *HDAC4* | -0.37428 | 0.001599 |
| *IFNA4* | 0.163477 | 0.000944 |
| *INTS3* | -0.34425 | 0.000576 |
| *K1AA1751* | 0.26881 | 0.000106 |
| *KDM3B* | -0.43558 | 0.001397 |
| *MAD2L1BF* | -0.27639 | 0.000721 |
| *MCTP1* | 0.550531 | 0.000158 |
| *MTRF1* | -0.30405 | 0.001865 |
| *NME7* | -0.53529 | 3.16E-05 |
| *NOTCH4* | 0.308965 | 0.001487 |
| *NUFIP1* | -0.39069 | 0.000295 |
| *OSGEPL1* | -0.25744 | 0.000868 |
| *PADI3* | 0.604017 | 0.001049 |
| *PAK4* | 0.343632 | 0.000244 |
| *PLXND1* | 0.281905 | 0.001885 |
| *POLR3C* | -0.38685 | 0.00175 |
| *PRKCE* | 0.172769 | 0.001832 |
| *RDH14* | -0.34108 | 0.001835 |
| *RIC8B* | -0.25297 | 0.00107 |
| *RSBN1* | -0.46581 | 0.000306 |
| *SERPINE2* | -0.94145 | 0.001831 |
| *SLC25A15* | -0.29922 | 0.001428 |
| *SLC30A3* | 0.253595 | 0.001541 |
| *SNRNP40* | -0.46085 | 0.000928 |
| *SPP2* | 0.164466 | 0.001187 |
| *SSR1* | -0.52779 | 0.000603 |
| *SSR3* | -0.52701 | 0.001781 |
| *STK38* | 0.214875 | 0.001148 |
| *SYN1* | 0.210248 | 0.000747 |
| *TCL1B* | 0.202839 | 0.001765 |
| *TFPI* | 0.471113 | 0.000521 |
| *TG* | 0.238306 | 0.001522 |
| *TMEM50B* | -0.43392 | 0.000815 |
| *TRIM13* | -0.43229 | 1.80E-06 |
| *TRMT1L* | -0.33956 | 0.001101 |
| *TTC30A* | -0.28427 | 0.000577 |
| *UBXN2B* | -0.3172 | 0.001 |
| *VIP* | 0.076004 | 0.00138 |
| *WSCD1* | 0.212376 | 0.001655 |
| *ZC3H13* | -0.44242 | 0.000147 |
| *ZFP36* | 0.399218 | 0.001181 |
| *ZNF193* | -0.3113 | 0.00186 |
| *ZNF710* | 0.219721 | 0.000643 |
| *ZNF79* | 0.13371 | 0.001752 |
| *1L1F9* | 0.161442 | 0.001741 |
| *201894_s_at* | -0.39129 | 0.000377 |
| *202225_at* | -0.44255 | 0.000112 |
| *214182_at* | 0.574394 | 0.000818 |
| *214912_at* | 0.65527 | 0.00022 |
| *215589_at* | 0.264618 | 0.00162 |
| *216170_at* | 0.330261 | 0.001332 |
| *216553_x_at* | 0.100986 | 0.000612 |
| *216658_at* | 0.140131 | 0.000902 |
| *216743_at* | 0.117028 | 0.001435 |
| *216783_at* | 0.273294 | 0.001086 |
| *219376_at* | -0.43479 | 0.000644 |
