# Supplementary material for: Comparison of molecular profile in triple-negative inflammatory and non-inflammatory breast cancer not of mesenchymal stem-like subtype
Source: PLoS One. 2019 Sep 18;14(9):e0222336. doi: 10.1371/journal.pone.0222336 (PMC6750603; doi:10.1371/journal.pone.0222336)
Supplement: S2 Table — (DOCX) [file pone.0222336.s003.docx]

**S2 Table. Eighty-one genes differentially expressed at an FDR of 0.4 between non-MSL TN-IBC and non-MSL TN-non-IBC based on Insight TNBCtype.**

| **Gene** | **Log2FC.IBC** | ***P* value** |
| --- | --- | --- |
| *AFFX-M278* | 0.582771 | 0.003258 |
| *ALMS1* | -0.44467 | 0.000189 |
| *ARL3* | -0.46892 | 0.00295 |
| *C2CD2L* | 0.272389 | 0.00342 |
| *C6orf124* | -0.43083 | 0.003717 |
| *C6orf162* | -0.40152 | 0.003071 |
| *C7orf44* | 0.646963 | 0.001863 |
| *CASP8AP2* | -0.47215 | 0.003511 |
| *CBL* | 0.190723 | 0.003038 |
| *CDC42EP3* | -0.75196 | 0.002207 |
| *COPG* | -0.55149 | 0.002524 |
| *DHX57* | -0.41118 | 0.000687 |
| *DOPEY1* | -0.50993 | 3.68E-06 |
| *ELF2* | -0.34965 | 0.001673 |
| *FFAR2* | 0.330057 | 0.003002 |
| *FPR1* | 0.538308 | 0.003244 |
| *GABPA* | -0.37852 | 0.0034 |
| *GLOD4* | -0.37969 | 0.003513 |
| *GTF2F2* | -0.26113 | 0.001444 |
| *IDE* | -0.35522 | 0.001968 |
| *INTS3* | -0.48698 | 1.00E-05 |
| *K1AA0907* | -0.42045 | 0.001253 |
| *K1AA1009* | -0.18888 | 0.002267 |
| *KDM1A* | -0.37434 | 0.001979 |
| *KDM3B* | -0.55905 | 0.000141 |
| *KIAA1751* | 0.296007 | 0.001234 |
| *LDC730102* | -0.68202 | 0.003328 |
| *LPIN2* | 0.496463 | 0.001037 |
| *LTN1* | -0.326 | 0.001716 |
| *MAPKAPK3* | 0.364162 | 0.002879 |
| *MCTP1* | 0.675077 | 3.45E-05 |
| *MDM4* | -0.32097 | 0.003353 |
| *MERTK* | 0.438852 | 0.000419 |
| *MICAL2* | 0.557805 | 0.002239 |
| *MTIF2* | -0.34615 | 0.003491 |
| *MTRF1* | -0.3563 | 0.003407 |
| *NCOR1* | -0.45342 | 0.000836 |
| *NFKBIL1* | -0.21287 | 0.003469 |
| *NUFIP1* | -0.46558 | 0.000462 |
| *ORC3* | -0.39449 | 0.003844 |
| *PAD13* | 0.750412 | 0.001086 |
| *PEX19* | -0.30969 | 0.00022 |
| *PHF3* | -0.34407 | 0.000928 |
| *PNRC1* | -0.30148 | 0.002842 |
| *PPOX* | -0.36735 | 0.000676 |
| *PRKD3* | -0.58415 | 0.00354 |
| *RCOR3* | -0.44793 | 0.001957 |
| *RFXAP* | -0.21529 | 0.002387 |
| *RIC8B* | -0.26655 | 0.000327 |
| *RPL34* | -0.30767 | 0.001349 |
| *RPL37A* | 0.669507 | 0.00369 |
| *RRNAD1* | -0.27499 | 0.002775 |
| *RSBN1* | -0.54819 | 0.000989 |
| *SAFB* | -0.33375 | 0.002734 |
| *SENP6* | -0.49573 | 0.000361 |
| *SLC13A1* | 0.057692 | 0.001981 |
| *SLC25A15* | -0.41117 | 0.000535 |
| *SOCS5* | -0.44766 | 0.003337 |
| *SSR1* | -0.65188 | 0.000819 |
| *TARDBP* | -0.60937 | 0.000936 |
| *TBC1D2* | 0.347851 | 0.002362 |
| *TCF4* | 0.671203 | 0.003782 |
| *TGM4* | 0.172291 | 0.00314 |
| *TPP2* | -0.44073 | 0.000381 |
| *TRIM13* | -0.47131 | 2.70E-05 |
| *TROVE2* | -0.41457 | 0.002765 |
| *UCN* | -0.28976 | 0.003606 |
| *ULK2* | -0.38295 | 0.003298 |
| *USP21* | -0.37399 | 0.000176 |
| *USPL1* | -0.39359 | 0.000962 |
| *VPS45* | -0.38382 | 0.003452 |
| *WRB* | -0.47367 | 0.001945 |
| *YPEL1* | -0.46452 | 0.003679 |
| *ZC3H13* | -0.6091 | 6.31E_05 |
| *ZNF702P* | 0.361538 | 0.001808 |
| *ZNF710* | 0.243535 | 0.003065 |
| *201894_s_at* | -0.50728 | 0.000264 |
| *202225_at* | -0.48548 | 0.000264 |
| *216170_at* | 0.399822 | 0.002772 |
| *219376_at* | -0.44625 | 0.003052 |
| *220874_at* | 0.16322 | 0.00321 |
